# Functional profiling of porcine precision-cut lymph node slices as an *ex vivo* model for TLR-mediated and PCV2-specific immune responses

**DOI:** 10.3389/fimmu.2026.1927543

**Published:** 2026-09-09

**Authors:** Samruddhi Deosthali, Bernat Marti-Garcia, Heather Harris, Natalie Werling, Robert Noad, Wilhelm Gerner, Dirk Werling

**Affiliations:** 1 Molecular Immunology Group, Centre for Vaccinology and Regenerative Medicine, Department of Pathobiology and Population Sciences, Royal Veterinary College, London, United Kingdom; 2 The Pirbright Institute, Woking, Surrey, United Kingdom

**Keywords:** cytokines, *ex vivo* model, germinal centre, PCV2, plasma cells, porcine lymph node slices, precision-cut tissue slices, R848

## Abstract

**Introduction:**

Precision-cut lymph node slices (PCLNS) provide a physiologically relevant ex vivo model to investigate lymphoid immune activation and vaccine adjuvant function while preserving native tissue architecture. In this study, we established for the first time porcine PCLNS and assessed the immunostimulatory capacity of PCLNS to Toll-like receptor (TLR) 7/8 agonist (Resiquimod; R848) alone or in combination with porcine circovirus type 2 (PCV2) or PCV2 virus-like particles (VLPs).

**Methods:**

Lymph nodes (LNs) were collected from PCV2-vaccinated pigs. PCLNS were generated using a Krumdieck tissue slicer and cultured in vitro with R848, PCV2, VLPs and their combinations Supernatants were collected daily for cytokine quantification and PCV2-specific antibody detection. Flow cytometry and immunofluorescence imaging were performed to examine B cell responses.

**Results:**

PCLNS maintained viability and preserved key histological structures over a five-day culture period, although progressive follicular depletion developed across conditions, potentially due to yet suboptimal culture condition. Phenotypic profiling revealed that R848 induces stronger B cell activation, including increased Ki-67 expression and plasma cell expansion. These cellular responses were accompanied by rapid and selective induction of IL-10, IL-12 and TNF following R848 stimulation. Daily supplementation with B cell survival factors (CD40L, BAFF and IL-21) failed to prevent germinal centre (GC) collapse despite modest effects on viability. Stimulation of PCLNS with PCV2 elicited detectable PCV2-specific IgG but not IgM, with the strongest humoral and cytokine responses observed following combined PCV2 and R848 stimulation, indicating a potential synergistic enhancement of recall-like immune response. In contrast, VLPs induced minimal antibody responses even with R848 co-stimulation. Collectively, these findings establish PCLNS as a tractable platform for assessing adjuvant-driven immune activation and demonstrate that TLR7/8 engagement seems to be a superior driver of B cell proliferation, plasma cell differentiation and pro-inflammatory cytokine production compared to other B cell survival factors, in porcine lymphoid tissue.

## Introduction

Precision-cut tissue slices (PCTS) constitute a robust, well-established *ex vivo* 3D culture platform that preserves native tissue architecture, cellular diversity, and intercellular communication networks. Since the introduction of the Krumdieck tissue slicer, PCTS have been widely applied across multiple organs and species to investigate pharmacological responses, toxicological mechanisms and host-pathogen interactions, thereby highlighting their broad applicability and versatility (1–19). Despite this extensive use, applications of PCTS derived specifically from lymph node (LN) remain limited. To date, precision-cut LN slices (PCLNS) have only been described in mice (20, 21) and humans (22, 23). Notably, Hoffmann and colleagues demonstrated that human LN and spleen derived PCTS retain sufficient microanatomical and cellular complexity to support spontaneous immunoglobulin (Ig) secretion and activation of both, B and T cells, indicating that essential immunological functions can be preserved *ex vivo* (22, 23). However, comparable systems have not been established in other species. Porcine PCLNS have not yet been reported, underscoring a notable gap in the current literature. Consequently, whether porcine LN derived PCTS can mount immune responses and produce Ig remains an avenue of exploration. This is particularly important given that LNs are central hubs for antigen presentation, lymphocyte priming and coordination of innate and adaptive immune responses. Thus, a porcine PCLNS platform capable of maintaining microanatomical and functional responsiveness would provide a valuable tool for dissecting antigen-specific immune responses *ex vivo*. In the present study, we describe the results of our experiments establishing porcine PCLNS as a physiologically relevant 3D *ex vivo* model for assessing innate and adaptive immune responses to a variety of model antigens. Using PCV2 and PCV2-VLP stimulation, we evaluated the system’s capacity to mount antigen-specific humoral immunity and investigated whether the TLR7/8 agonist R848 can enhance these responses similar as described in other species (24–28). We believe that porcine PCLNS can provide a more appropriate system compared to studies performed in mice. While these can be infected with PCV2 via the oral or intra-peritoneal route, they do not show development of the classical clinical symptoms or overt systemic disease seen in pigs (such as severe postweaning multisystemic wasting syndrome, marked respiratory distress, or high mortality). Furthermore, the PCV2 ORF3 protein has very limited pathogenicity in its primary host, whereas it seems to play a prominent role in the murine models (29, 30). Thus, in the context of performing experiment from the beginning on within the target species, and taken the 3R principles into account, we believe that PCNLS are better suited compared to the murine system.

## Materials and methods

### Ethics statement and animals

This project has been ethically reviewed and approved by the Clinical Research Ethical Review Board (CRERB) of the Royal Veterinary College (URN number: 2023 2179-2). LNs were taken from 6- to 10-month-old male and female P81 pigs (high-performance commercial sire line developed by Choice Genetics, now part of Axiom/Klasse AI) humanely dispatched in a commercial slaughterhouse. All animals were clinically healthy and declared fit for human consumption by the Food Standards Agency, UK appointed veterinarians. LNs obtained were from animals vaccinated against PCV2 at 3 to 4 weeks of age. Mandibular, retropharyngeal and superficial cervical LNs were collected into phosphate-buffered saline (PBS) supplemented with penicillin-streptomycin (P/S; 100 U mL^-1^; Gibco, 15140122) and amphotericin B (2.5 µg mL^-1^; Gibco, 15290018).

### Preparation of PCLNS and experimental design

**Figure 1:**
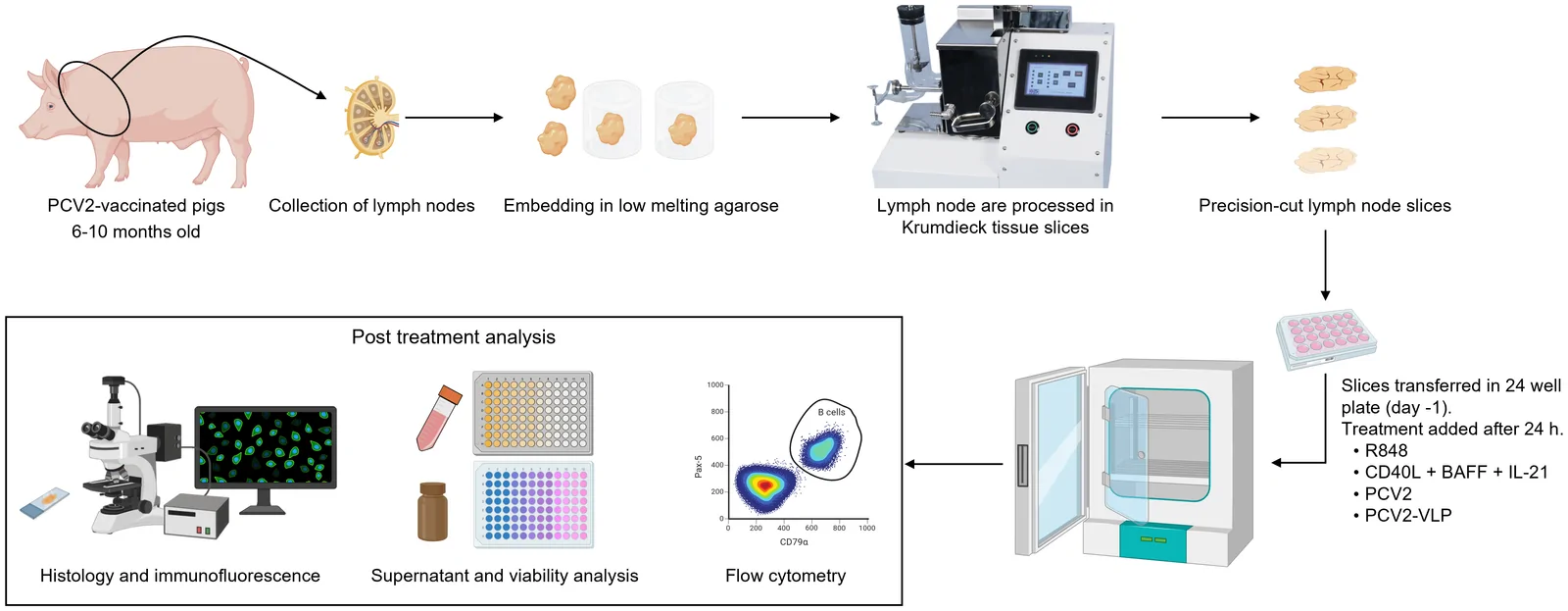
Workflow for generation and analysis of PCLNS derived from porcine LN.

**Figure 2:**
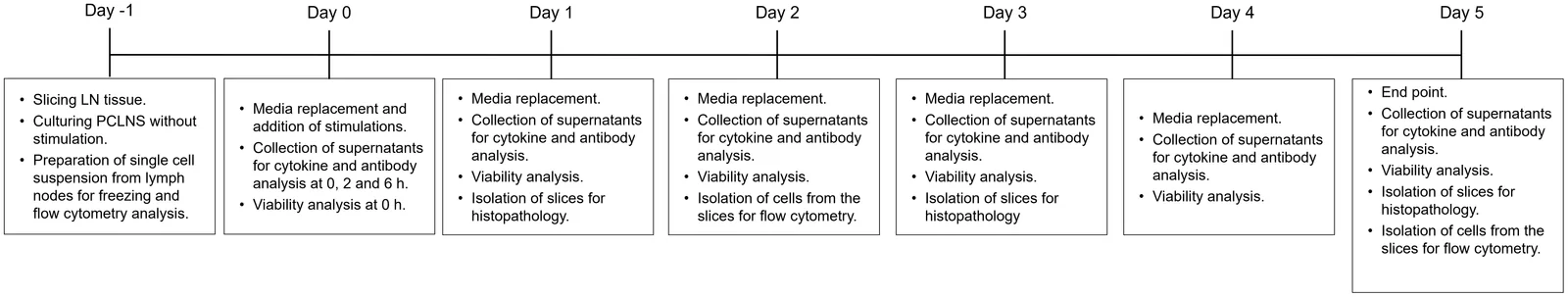
Schematic representation of PCLNS experimental timeline.

Upon arrival, adipose tissue surrounding the LNs was carefully removed using micro-surgical scissors, sectioned into smaller fragments and transferred into a core holder (**Figure 1**). The gaps between the tissue and core holder were filled with warm 1.5% low-melting-point agarose (Merck, A0701-25G). The prepared core holder was placed in a Krumdieck Tissue Slicer (Alabama Research and Development, MD6000), filled with cold PBS containing P/S and amphotericin B and attached to a cooling pump set. Obtained PCLNS of 300 µm in thickness were collected in a container with PBS supplemented by P/S and Amphotericin B and then transferred to a 24-well tissue culture plate (one slice per well) containing 1 mL of pre-warmed RPMI GlutaMax medium supplemented with 10% FBS, P/S, amphotericin B, and Insulin-Transferrin-Selenium-Ethanolamine (ITS-X, 100x; Gibco, 51500056) and incubated at 38.5 °C. The day of tissue acquisition and PCLNS preparation was designated as day -1 during which the slices were left for 24 h in media alone (**Figure 2**). In parallel, porcine LNs were processed to prepare a single cell suspension for flow cytometric analysis and cryopreservation. On day 0, PCLNS were transferred into fresh 24-well plates containing pre-warmed media and different stimulations. Slices were stimulated with one or more of the following factors, depending on the experimental setup: R848 (2.5 μg mL^-1^; StemCell Technologies, 73782), recombinant human CD40L [1 μg mL^-1^; BioLegend, 591706, which cross-reacts on porcine cells (31)], porcine B-cell activating factor (BAFF; 50 ng mL^-1^; Kingfisher Biotech, RP0492S-025), recombinant porcine IL-21 (10 ng mL^-1^; Kingfisher Biotech, RP0937S-025), and media was replenished daily. All factors have been shown to be active before (32–34). Furthermore, PCLNs were stimulated using PCV2b derived from a molecular clone (35). Slices were incubated with 1.5 x 10^6^ PCV2b DNA copies per slice (prepared as described (35)), or purified and adjuvant-free PCV2a-ORF2 VLPs (approximately 1.5 x 10^6^ PCV2-VLPs per slice, provided by Boehringer Ingelheim Animal Health USA Inc.). This approach was chosen since biologically, a single PCV2 VLP/capsid packages precisely one single-stranded circular DNA genome (~1.7 kb), establishing a strict theoretical 1:1 structural stoichiometry for assembled mature virions (36). Empty capsids or imperfect assembly intermediates during recombinant over-expression were removed in the current experiments due to the purification process during vaccine generation. Tissue viability was monitored using the PrestoBlue™ Cell Viability assay from day 0 to 5 post stimulation (**Figure 2**). Culture supernatants were harvested at 2 h and 6 h, thereafter, daily to assess cytokine and PCV2-specific antibody production. For structural and phenotypic analysis, PCLNS were either embedded in optimal cutting temperature (OCT) compound or fixed in formalin (4% v/v) at day 0, 1,3 and 5 for immunofluorescence and histology respectively. Finally, cells were isolated from PCLNS on day 2 and 5 for flow cytometric phenotyping of B and T cell.

### Viability analysis of PCLNS

Viability of PCLNS was assessed daily from day 0 to day 5 using the Prestoblue™ Cell Viability Reagent (Invitrogen, A13262) according to manufacturer’s recommendation (**Figure 2**). While this dye measures mainly metabolic activity as a marker for viability, we will use the term “viability” for sake of simplicity in this manuscript. Briefly, Prestoblue reagent was added to the wells containing PCLNS and incubated in the dark for 45 mins at 38.5 °C in an incubator. Fluorescence intensity was measured using a Tecan Infinite 200 PRO Plate Reader (Tecan Instruments) at 560/590 nm excitation/emission wavelengths.

### Histological preparation and analysis of PCLNS

For haematoxylin and eosin (H&E) staining, PCLNS were wrapped in bio-wrap (Leica, 3801090), placed in a fixation cassette and submerged in 4% formalin. H&E staining on formalin fixed slices was performed using a Leica Autostainer XL (ST5010; RVC Pathology service). Histological analysis of H&E-stained PCLNS was performed using QuPath (version 0.5.1). To quantify lymphoid follicular area, a representative area of 3–4 mm^2^ was selected and annotated on each section (region of interest; ROI). Within each ROI, individual follicular areas were measured, and mean values were calculated for comparative analysis. The total number of follicles were quantified manually across the entire surface area of each PCLNS. Structural alterations within the follicular and paracortical regions were categorised as normal, hyperplastic or depleted based on the following defined histological criteria:

1. Follicular evaluation: Follicular structures were evaluated based on their morphological integrity within the cortex.

- Normal follicle: Characterised by round, uniform and densely populated lymphocytes lacking pale germinal centres (GCs).
- Hyperplastic follicle: Recognised by enlarged or coalescing follicles containing prominent, paler-staining GCs surrounded by a tightly packed mantle zone.
- Depleted or atrophied follicle: Identified by reduced lymphocyte density, exposing the underlying stroma and absent GCs accompanied by thinned or collapsed mantle zones.

2. Paracortical evaluation: The interfollicular structures were evaluated based on cellular density and structural morphology.

- Normal paracortex: Composed of tissue surrounding the follicles and medulla, populated by distribution of lymphocytes, macrophages and DCs.
- Hyperplastic paracortex: Characterised by a diffuse increase in cellular density alongside expansion of the paracortical areas.
- Depleted or atrophied paracortex: Identified by reduced cellular density, loss of lymphocytes and visibility of the underlying stromal cells and structural architecture.

### Isolation of cells and B cell staining strategy

On days −1, 2, and 5, LN or PCLNS tissue slices were mechanically dissociated by pressing tissue through a 70 μm strainer with a syringe plunger, followed by two cold PBS washes and two additional passes through a 70 μm strainer to generate single-cell suspensions. Cells were transferred to 96-well U-bottom plates and stained using a multistep protocol for extracellular and intracellular markers. For surface staining, cells were incubated with CD3ϵ and Fixable Viability Dye eFluor 780 (Thermo Fisher Scientific, 65-0865-14) to exclude dead cells (**Table 1**). Afterward, cells were fixed and permeabilised using the FoxP3/transcription factor staining buffer set (Thermo Fisher Scientific, 00-5523-00) and stained for intracellular markers including CD79α, Pax-5, Blimp-1, IRF4, Bcl-6, Ki-67, and IgM as described (37). All anti-human, mouse, and rat antibodies were validated for cross-reactivity in pigs and titrated in single-colour controls to optimise signal-to-noise ratios (37). CD79α was used for parent gating strategy, with the percentages derived for other B cell subsets being from the CD79^+^ Pax-5^+^/low population (37) (See **Supplementary Figure 1** for gating strategy). Samples were acquired on a BD Fortessa (UV 355 nm, Violet 405 nm, Blue 488 nm, Red 640 nm) or a Cytek Aurora (UV 355 nm, Violet 405 nm, Blue 488 nm, Yellow-Green 561 nm, Red 640 nm), and data were analysed using FlowJo v10.10.0 (BD Biosciences).

**Table 1:** Antibodies and reagents used for flow cytometry and immunofluorescence.

Antigen	Clone	Isotype	Fluorochrome	Labelling strategy	Source
CD3ϵ	PPT3	mouse IgG1	FITC	directly conjugated	Bio-Rad
CD79α	HM47	mouse IgG1	PerCP-Cy5.5	directly conjugated	BioLegend
			APC		
Pax5	1H9	rat IgG2a	PE-CF594	directly conjugated	BD Biosciences
Blimp-1	3H2-E8	mouse IgG1	AF647	directly conjugated	Santa Cruz Biotech
IRF4	3E4	rat IgG1	PE	directly conjugated	Thermo Fisher Scientific
Bcl6	K112-91	mouse IgG1	BV421	directly conjugated	BD Biosciences
Ki-67	B56	mouse IgG1	BUV395	directly conjugated	BD Biosciences
IgM	polyclonal	Goat	Biotinylated	secondary antibody^1^	Bio-Rad
^1^Streptavidin			BV605	directly conjugated	BD Biosciences

### Quantification of cytokines

#### Porcine multiplex immunoassay

Secreted IL-1β, IL-4, IL-6, IL-8, IL-10, IL-12, IFN-α, IFN-γ and TNF was determined using either the ProcartaPlex™ cytokine & chemokine panel 1–9 plex (Invitrogen, EPX090-060829-901) according to the manufacturer’s instructions or the Porcine Luminex^®^ Discovery Assay (R&D Systems Inc, LXSAPM-08) according to the manufacturer’s instructions. Briefly, supernatants were harvested and plates were analysed using a Bio-Plex™ system (Bio-Rad). Concentrations of each cytokine were calculated by plotting expected concentration of the standards against median fluorescence intensity (MFI) and generating a standard curve for each analyte using a 5-parameter logistic (5-PL) curve-fit (Bio-Plex™ Manager software, version 6.2). The final concentrations of each analyte were plotted on GraphPad Prism software (GraphPad Inc, version 10.4.2).

### ELISA for porcine IL-12 and TNF

For a targeted assessment, IL-12 and TNF were measured using the porcine DuoSet ELISA development kits (R&D Systems Inc; DY912 and DY690B respectively) according to manufacturer’s instruction. Plates were measured using a microplate photometer (MultiSkan FC, Thermo Fisher Scientific) set to 450 nm using Thermo Scientific SkanIt™ software. Both, the standard curve and the values obtained for each cytokine in pg mL^-1^ were plotted on graphs (GraphPad Prism).

### ELISA for PCV2 specific IgG and IgM

PCV2-specific IgG and IgM antibodies were measured using the Ingezim^®^ Circovirus IgG/IgM ELISA kit (Gold Standard Diagnostics, 11.PCV.K2), according to the manufacturer’s protocol. Absorbance was measured at 450 nm on a microplate photometer (MultiSkan FC, Thermo Fisher Scientific) using Thermo Scientific SkanIt™ software. To standardise values across experiments, responses were expressed as the ratio of sample OD to positive control OD and plotted using GraphPad Prism.

### Immunofluorescence staining of PCLNS

PCLNS were embedded in OCT Embedding Matrix and 10 µm sections of OCT embedded tissue were obtained using Bright OTF5000 Cryostat (Bright Instruments Company). Once the OCT was dried, the sections were fixed in cold 99.8% acetone for up to 20 min. The slides were incubated with 5% BSA in PBS, followed by overnight incubation with primary antibodies (**Table 1**). The following day, the slides were washed with PBS + 0.05% Tween 20. For staining with DAPI and F-actin staining, 50 µL of 4′,6-diamidino-2-phenylindole (DAPI; VectaShield^®^ HardSet™ Antifade Mounting with DAPI, H-1500-10) and 50 µL Phalloidin (VectaShield^®^ HardSet™ Antifade Mounting Medium with Phalloidin, H-1600) were combined and added on to the slides. A glass coverslip was mounted on the top of the glass slide and images were acquired using Nikon Fluorescent Microscope.

### Statistical analysis

Where appropriate, data were subsequently analysed using GraphPad Prism software 10.4.2. PCLNS stained with H&E were assessed utilising the framework described by (38).

## Results

### Activating TLR7/8 via R848 supports structural integrity viability of PCLNS

**Figure 3:**
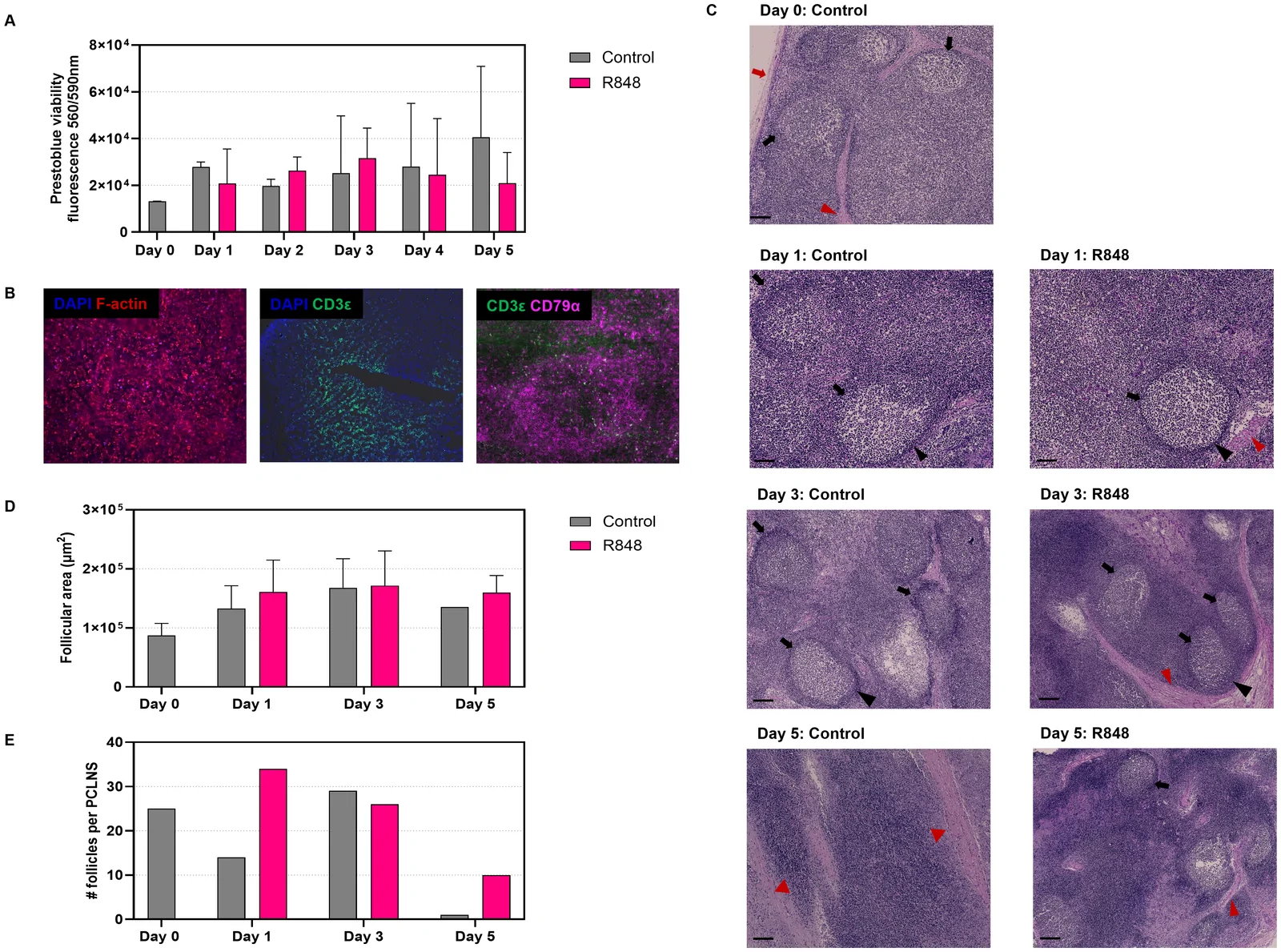
Viability and maintenance of tissue architecture in PCLNS during 5-day culture following stimulation with R848. **(A)** Viability of PCLNS was monitored from days 0 to 5 following treatment with R848 or control. Metabolic viability was assessed using a resazurin-based assay. Following a 45 min incubation with resazurin, culture supernatants were collected and resorufin fluorescence was measured at excitation/emission wavelengths of 560/590 nm. **(B)** Representative immunofluorescence images of PCLNS stained with DAPI and F-actin (left), DAPI and CD3ϵ (centre), and CD3ϵ and CD79a (right). DAPI is shown in blue, F-actin and CD79a in magenta and CD3ϵ in green. **(C)** Representative H&E-stained sections illustrating PCLNS morphology at day 0, 1, 3 and 5, either left untreated or following treatment with R848. Annotated anatomical features include lymphoid follicles (black arrow), the mantle zone (black arrowhead), connective tissue trabeculae (maroon arrowhead) and the capsule (maroon arrow). Scale bar = 0.5 cm. **(D, E)** Morphometric analysis of lymphoid follicles in H&E-stained PCLNS. **(D)** Quantitative analysis of follicular area (µm²) from days 0 to 5 in control and R848-treated PCLNS. **(E)** Total number of follicles identified in PCLNS over the 5-day culture period. Data are presented as mean ± SD. For the viability assay, *n* = 2 biological replicates were measured in technical duplicates, with each biological replicate representing the mean of two duplicate PCLNS. Statistical significance was determined using two-way repeated-measures ANOVA followed by Tukey’s multiple comparisons test for viability assay and morphometric date with the day 0 baseline timepoint excluded from the analysis. For follicular area, means were calculated from multiple follicles within predefined ROI from a single biological replicate (*n* = 1).

PCLNS were stimulated with R848 to assess immune responsiveness. Metabolically active cells within the PCLNS as a correlate of the cell viability was measured using a resazurin-based assay. In both control and R848 stimulated slices, viability increased from day 0 to day 1 (**Figure 3A**). In control slices, viability remained stable until day 2, then became more variable from day 3 and peaked on day 5 (**Figure 3A**). In contrast, R848-treated slices maintained consistent metabolic activity from day 1 through day 5 (**Figure 3A**). In addition to assessing viability, we also assessed whether viability data could be corroborated by morphological changes. To do so, slices were stained for T cells and B cells by H&E and immunofluorescence. Using immunofluorescence to visualise the lymphoid architecture of PCLNS, T cell and B cell compartments were clearly distinguishable. PCLNS sections were stained with DAPI and F-actin to assess cellular structure or with CD79α or CD3ϵ to label B and T cell areas, respectively (**Figure 3B**). These markers enabled clear delineation of distinct B and T cell regions within the slices. Furthermore, H&E stain showed that on day 0, nodal architecture of PCLNS was well preserved, featuring an intact outer capsule (maroon arrow) and connective tissue trabeculae (maroon arrowhead), without any evidence of architectural effacement, lymphocytolysis, haemorrhage or oedema (**Figure 3C**). Numerous, lymphoid follicles (black arrow) were hyperplastic, featuring prominent pale GCs surrounded by concentric mantle zones (black arrowhead) (**Figure 3C**). Throughout the sections, follicles and paracortex primarily comprised small lymphocytes (4-6 µm in diameter) interspersed with some histiocytes, remaining identifiable until day 5 in some treatments. In control sections on days 1 and 3, follicles appeared enlarged, hyperplastic and coalescing alongside a normal paracortex, whereas by day 5, the follicles were completely absent and accompanied by a normal to depleted paracortex. R848 stimulated slices demonstrated follicular hyperplasia and a normal paracortex on day 1, followed by both follicular and paracortical hyperplasia on day 3, eventually leading to fewer hyperplastic follicles with pronounced paracortical hyperplasia on day 5. In line with these histological alterations, morphometric analysis of lymphoid follicles revealed shifts in both size and frequency throughout the culture period (**Figures 3D, E**). Compared to the day 0 baseline, follicular areas exhibited an increase across all treatments on days 1, 3 and 5 (**Figure 3D**). Follicular areas in the control and R848 treatments remained comparable and stable from day 1 to day 5. Conversely, follicular numbers per slice were higher in R848 stimulated sections compared to control on day 1 (**Figure 3E**). Control and R848 treated groups were consistent with each other on day 3, before the numbers progressively declined in both groups by day 5, with a more pronounced reduction observed under control conditions.

### Targeting TLR7/8 via R848 induces plasma cell proliferation and cytokine responses in PCLNS

**Figure 4:**
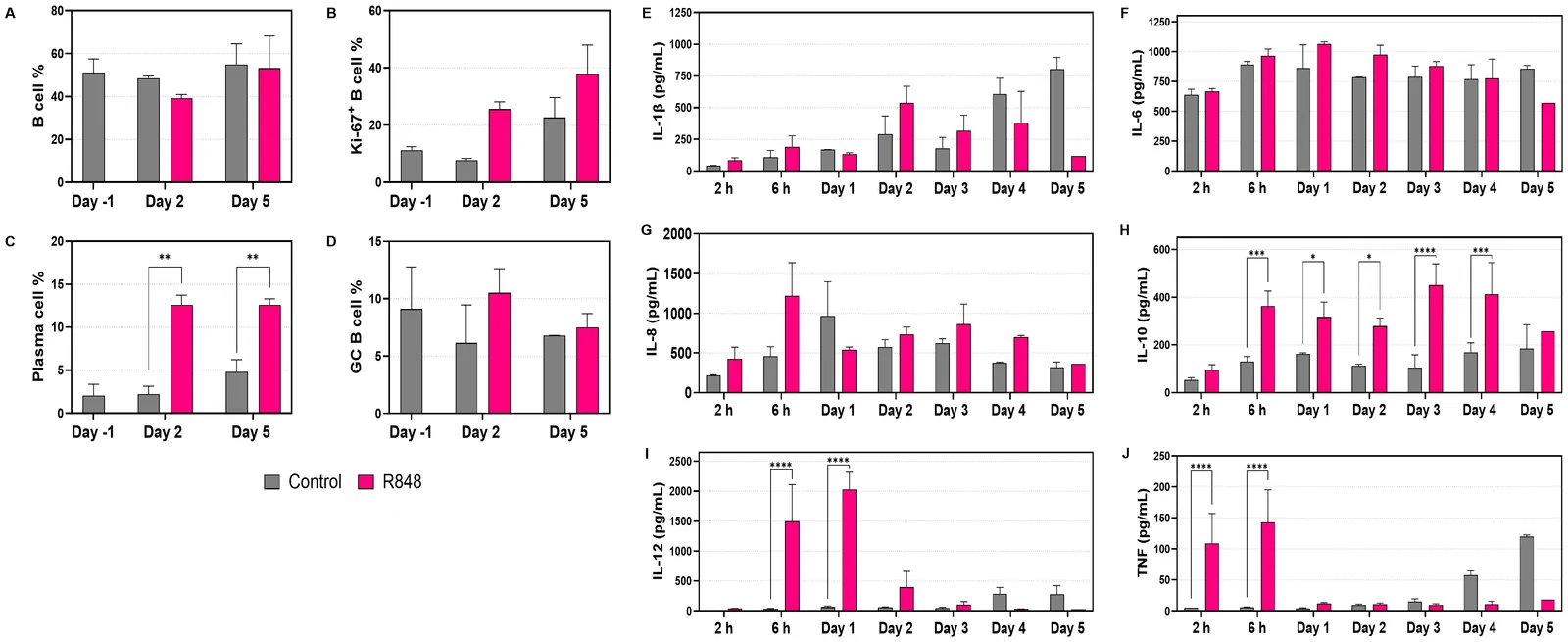
R848 stimulation induces B cell phenotypic changes and cytokine production in porcine PCLNS. **(A–D)** Percentages of B cell subsets in porcine PCLNS following stimulation with R848 or control. Cell populations were quantified on day −1 (slicing day; derived by preparing single cell suspension from porcine LNs), day 2, and day 5. **(A)** Total B cells, **(B)** Ki-67^+^ B cells, **(C)** Plasma cells and **(D)** GC B cells. All populations were identified by sequential gating on forward scatter area (FSC-A), side scatter area (SSC-A), singlets, live cells and the indicated B cell subsets (see **Supplementary Figure 1**). **(E–J)** Time-course analysis (2 h to day 5) of cytokine concentrations in culture supernatants from PCLNS stimulated with R848 or control. Concentrations of **(E)** IL-1β, **(F)** IL-6, **(G)** IL-8, **(H)** IL-10, **(I)** IL-12 and **(J)** TNF are expressed as pg mL^-1^. Data are presented as mean ± standard deviation (SD) from *n* = 2 biological replicates, with each biological replicate representing the mean of two duplicate PCLNS, each analysed in technical duplicate. Statistical analyses were performed using two-way repeated-measures ANOVA, followed by Tukey’s multiple comparisons test. For B cell phenotyping, the day −1 baseline time point was excluded from the ANOVA model. Statistical significance is indicated as *p ≤ 0.05, **p ≤ 0.01, ***p ≤ 0.001, ****p ≤ 0.0001.

In parallel with these morphometric changes, phenotypic analysis of R848 stimulated PCLNS showed stability of total B cells up to day 5 (**Figure 4A**; for detailed B cell gating strategy see **Supplementary Figure 1**). Percentage of Ki-67^+^ B cells increased at day 2 with R848 stimulation, followed by further elevation on day 5 (**Figure 4B**). Within total B cells, the percentage of plasma cells was low and comparable in unstimulated samples from day -1 to day 5 (**Figure 4C**), while R848 stimulation induced significant expansion of plasma cells on both, day 2 and 5 (**Figure 4C**). On day 2, GC B cell proportions in the control group remained comparable to day -1 baseline levels, while a slight increase was observed following R848 stimulation (**Figure 4D**). By day 5, these proportions were similar across all treatment groups, remaining marginally below the baseline. In addition to this, a small fraction of cells had migrated from the slices into the surrounding culture wells and were assessed separately to determine whether the migrating cells exhibited phenotypic differences (**Supplementary Figure 1**). While there was no major phenotypic divergence, R848 stimulation promoted B cell, plasma cell and GC B cell proliferation.

As changes in B cells/plasma cell composition are often accompanied by changes in cytokine release, we next evaluated whether R848 stimulation of PCLNS also impacted the overall cytokine profile. Overall, detectable levels for IL-1β, IL-6, IL-8, IL-10, IL-12 and TNF were seen, while IL-4, IFN-α and IFN-γ remained below detection limits (**Figures 4E–K**). Among the detectable cytokines, IL-10, IL-12 and TNF exhibited treatment-specific induction.

IL-1β increased non-selectively from day 2 onwards with minimal variability between treatments (**Figure 4E**), while IL-6 remained consistent across all timepoints and treatments (**Figure 4F**). IL-8 increased transiently at 6 h following R848 stimulation and on day 1 in control slices but remained comparable across all conditions from day 2 to day 5 (**Figure 4G**). In contrast, IL-10 was significantly elevated at 6 h and sustained until day 4 in R848 stimulated PCLNS (**Figure 4H**). The pro-inflammatory cytokines IL-12 and TNF displayed early and transient induction following stimulation with R848 (**Figures 4I, K**). IL-12 increased significantly at 6 h and day 1 before declining from day 2 to day 5, while TNF peaked significantly at 2 and 6 h and decreased from day 1 onwards. In controls, IL-12 and TNF remained low, although TNF showed a moderate late increase on days 4 and 5.

### B cell survival factors fail to rescue GC responses in PCLNS

Given the reduction in lymphoid follicles and GCs frequency evidenced by H&E staining and flow cytometry, supplementation with CD40L, BAFF and IL-21 (collectively termed CBI) was evaluated as a strategy to preserve follicular and GCs architecture, enhance B cell survival and support vaccine adjuvant application, similar as described before (34, 39, 40).

**Figure 5:**
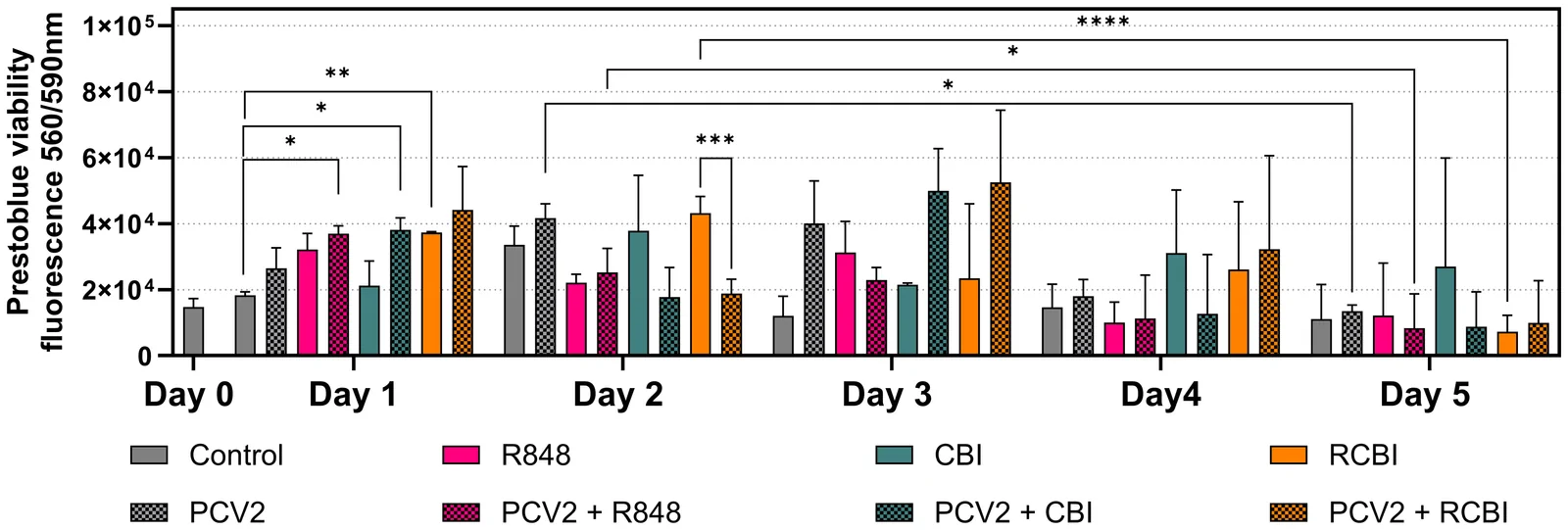
Viability of PCLNS during 5 days of culture following stimulation with R848, B cell survival factors, porcine circovirus type 2 (PCV2) and their combinations. Metabolic viability of porcine PCLNS was monitored from days 0 to 5 following treatment with R848, the B cell survival factors (CD40L, BAFF and IL-21; collectively referred as CBI in the graphs), PCV2 or their combinations. Viability was assessed using a resazurin-based assay. Following a 45 min incubation with resazurin, culture supernatants were collected and resorufin fluorescence was measured at excitation/emission wavelengths of 560/590 nm. Data are presented as mean ± SD from *n* = 3 biological replicates, with each biological replicate representing the mean of two duplicate PCLNS, each analysed in technical duplicate. Statistical analysis was performed using two-way repeated-measures ANOVA, excluding the day 0 baseline time point, followed by Tukey’s multiple comparisons test. Statistical significance is indicated as *p ≤ 0.05, **p ≤ 0.01, ***p ≤ 0.001, ****p ≤ 0.0001.

Metabolically active cells within the PCLNS stimulated with combinations R848, CBI and PCV2 combinations were measured as a correlate of the cell viability (**Figure 5**). Consistent with previous observations, cell viability increased across all experimental conditions from 0 h to day 1. On day 1, all treatments exhibited higher viability than control, with PCV2 + R848, PCV2 + CBI and R848 + CBI (RCBI) stimulations reaching statistical significance. On day 2, viability (compared to controls at day 0) remained elevated in controls, PCV2, CBI and RCBI groups, while declining in R848, PCV2 + R848, PCV2 + CBI, and PCV2 + RCBI conditions. On day 3, viability in controls decreased, whereas PCV2, PCV2 + CBI and PCV2 + RCBI groups reached their peak viability. A general reduction in the viability was observed across all treatment groups from days 4 to 5.

**Figure 6:**
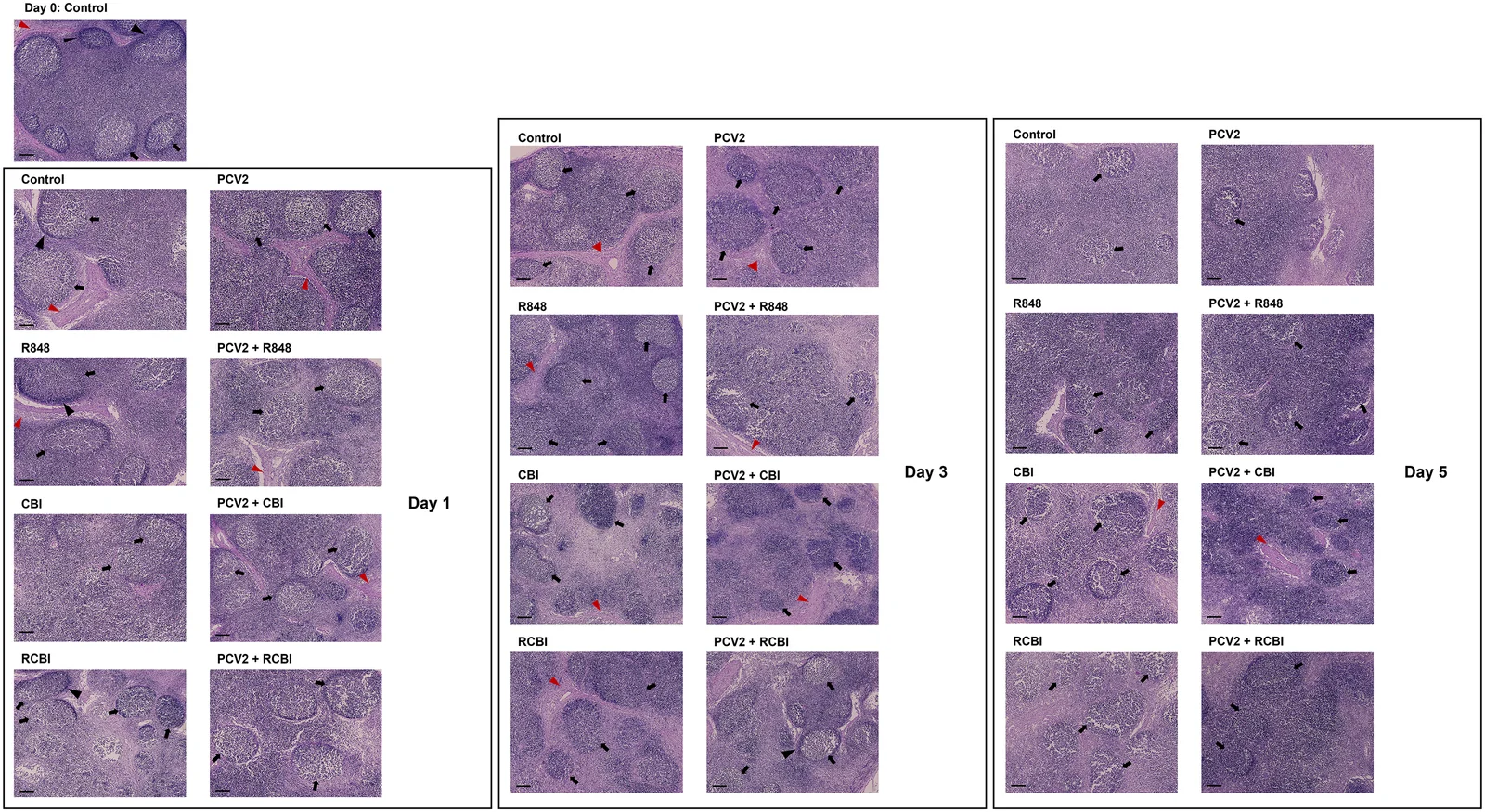
Histological analysis of PCLNS integrity and morphology following stimulation with R848, CD40L + BAFF + IL-21 (CBI), PCV2 and their combinations. Representative H&E-stained sections illustrating PCLNS morphology at day 0, 1, 3, and 5 following stimulations in combination of R848, CBI, PCV2 and control. Annotated anatomical features include lymphoid follicle (black arrow head), mantle zone (black arrow) and connective tissue trabeculae (maroon arrowhead). Scale bar= 0.5 cm).

Histological examination of PCLNS further elucidated the progression of lymphoid architectural changes over time induced by R848, CBI and PCV2 combinations (**Figure 6**). On day 0, the well-preserved LN architecture featured intact, hyperplastic lymphoid follicles (black arrow) with pale GCs and concentric mantle zones (black arrowhead) supported by fibrous trabeculae (maroon arrowhead). Follicular and parafollicular regions comprised small lymphocytes (4-6 µm) along with histiocytes, all persisting until day 5. Control slices on day 1 revealed hyperplastic follicles with prominent GCs and a normal paracortex that transitioned to normal follicles by day 3 and subsequently demonstrated mild follicular depletion and a normal paracortex on day 5. Conversely, PCV2 and PCV2 + R848 treatments followed a shared trajectory where day 1 follicular hyperplasia and a normal paracortex progressed on day 3 to normal follicles or a mild loss of follicular definition alongside paracortical depletion, progressing by day 5 to normal to mildly depleted follicles and paracortex. Similarly, R848 and PCV2 + RCBI stimulated groups both maintained hyperplastic follicles on days 1 and 3, shifting by day 5 to slightly depleted follicles with a normal to hyperplastic paracortex. For the CBI and PCV2 + CBI groups, day 1 hyperplastic follicles and a normal paracortex progressed on day 3 to normal follicles with a depleted paracortex, culminating on day 5 in normal, densely populated follicles alongside a slightly hyperplastic paracortex. Finally, RCBI stimulated slices displayed degradation by day 5, where day 1 hyperplastic follicles and a marginally depleted paracortex transitioned on day 3 to normal follicles with densely populated lymphocytes and a depleted paracortex, before demonstrating a loss of follicular architecture and a depleted paracortex by day 5.

**Figure 7:**
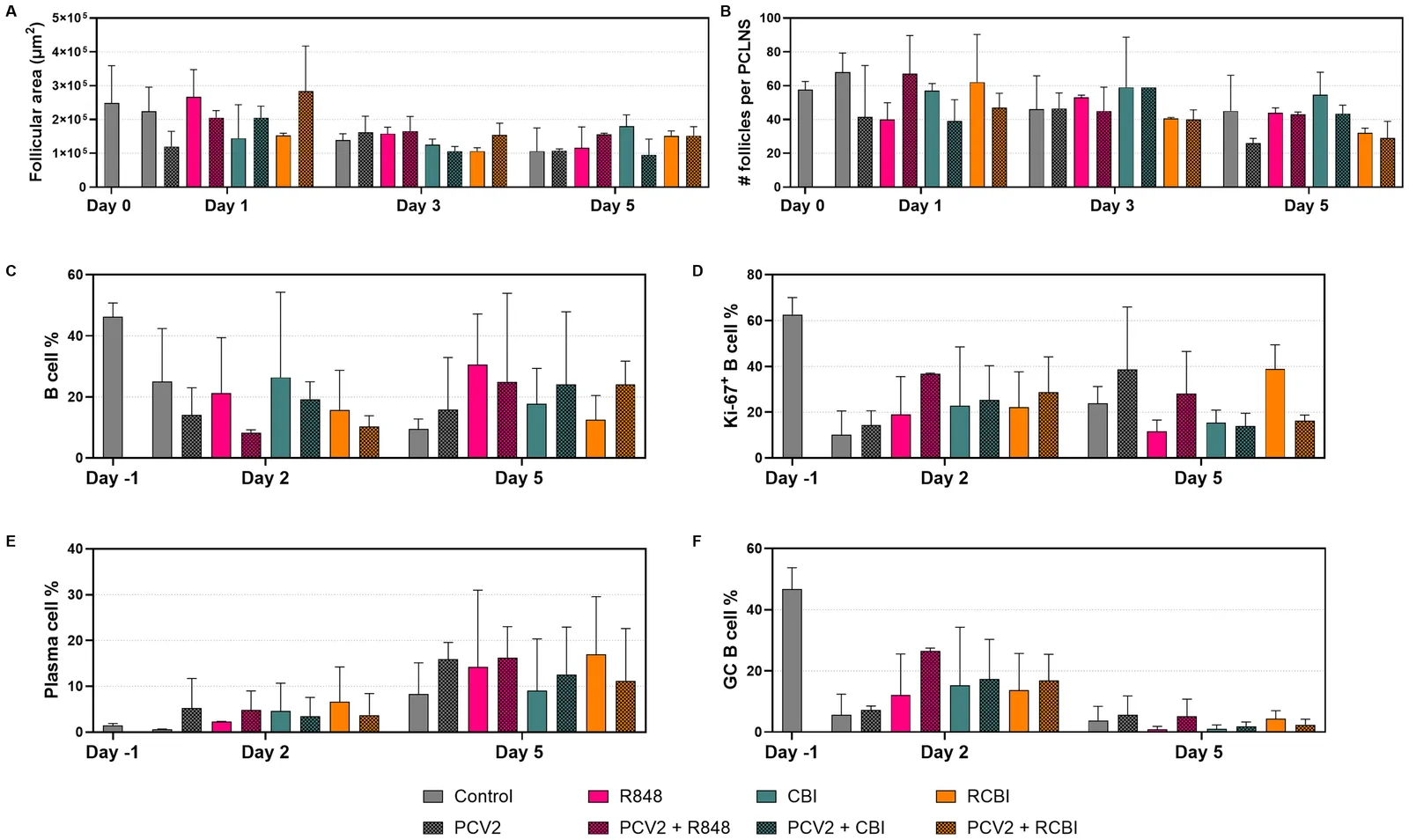
Morphometric analysis of lymphoid follicles and B cell phenotypes in PCLNS following stimulation with R848, CBI, PCV2 and their combinations. **(A)** Quantitative analysis of follicular area (µm²) in PCLNS from days 0 to 5 following treatment with R848, CBI, PCV2 or their combinations. Data are presented as mean ± SD of multiple follicles within predefined regions of interest in PCLNS (Section materials and methods- Histological preparation and analysis of PCLNS) **(B)** Total number of follicles identified in PCLNS over the 5-day culture period. **(C–F)** Percentages of B cell subsets in porcine PCLNS at day −1 (derived from fresh LN single-cell suspensions), day 3 and day 5 following stimulation with R848, CBI, PCV2 or their combinations. Populations analysed include **(C)** total B cells, **(D)** Ki-67^+^ B cells, **(E)** plasma cells and **(F)** GC B cells. All subsets were identified by sequential gating on FSC-A versus SSC-A, singlets, live cells and the indicated B-cell populations. Data are presented as mean ± SD of *n* = 3 biological replicates. For follicular area, means were calculated from multiple follicles within ROI per treatment and timepoint across *n* = 3 biological replicates. Statistical significance was determined by two-way repeated-measures ANOVA followed by Tukey’s multiple comparisons test. The day 0 timepoint was excluded from the morphometric analysis and the day −1 timepoint was excluded from the B cell phenotyping analysis.

Morphometric analysis of H&E stained PCLNS quantified these architectural changes, showing an overall downward trend in follicular size over time (**Figure 7A**). Mean follicular area decreased from day 0 to day 3 and remained reduced through day 5. Follicular areas were largest in PCV2 + RCBI, followed by R848 stimulated cohorts on day 1. Beyond this early variation, follicular size was broadly comparable across treatments from day 3 to day 5. While follicle number remained stable from day 0 to day 5, all treatment groups exhibited a modest decline in follicular count by day 5, although the overall range stayed comparable to earlier timepoints (**Figure 7B**).

Phenotypically, B cell proportions declined from day 2 to day 5 (**Figure 7C**). On day 2, CBI treated slices exhibited the highest B cell proportions, followed by control and R848, whereas all PCV2 treated slices (± adjuvants) had lower B cell proportions than control and their corresponding adjuvant-only stimulations. In contrast, on day 5, B cell proportions were highest in R848 stimulated slices, followed by PCV2 + R848, PCV2 + CBI and PCV2 + RCBI. Ki-67^+^ B cells were overall reduced on day 2, with the highest levels observed in PCV2 + R848 group and otherwise remained comparable across treatments (**Figure 7D**). On day 5, Ki-67^+^ B cells were most abundant in PCV2 and RCBI stimulated PCLNS, followed by PCV2 + R848 and control. Plasma cell proportions increased from day -1 to day 5 in all treatment cohorts, peaking on day 5 in PCV2, R848, PCV2 + R848 and RCBI stimulated conditions, while remaining low but comparable in the other cohorts (**Figure 7E**). GC B cells declined sharply from day -1 to day 5 in all treatment groups (**Figure 7F**). While levels were highest in PCV2 + R848 condition on day 2, those in other treatments remained broadly comparable, before becoming depleted across all conditions by day 5.

### B cell survival factors do not seem to impact on cytokine and antigen-specific antibody responses

**Figure 8:**
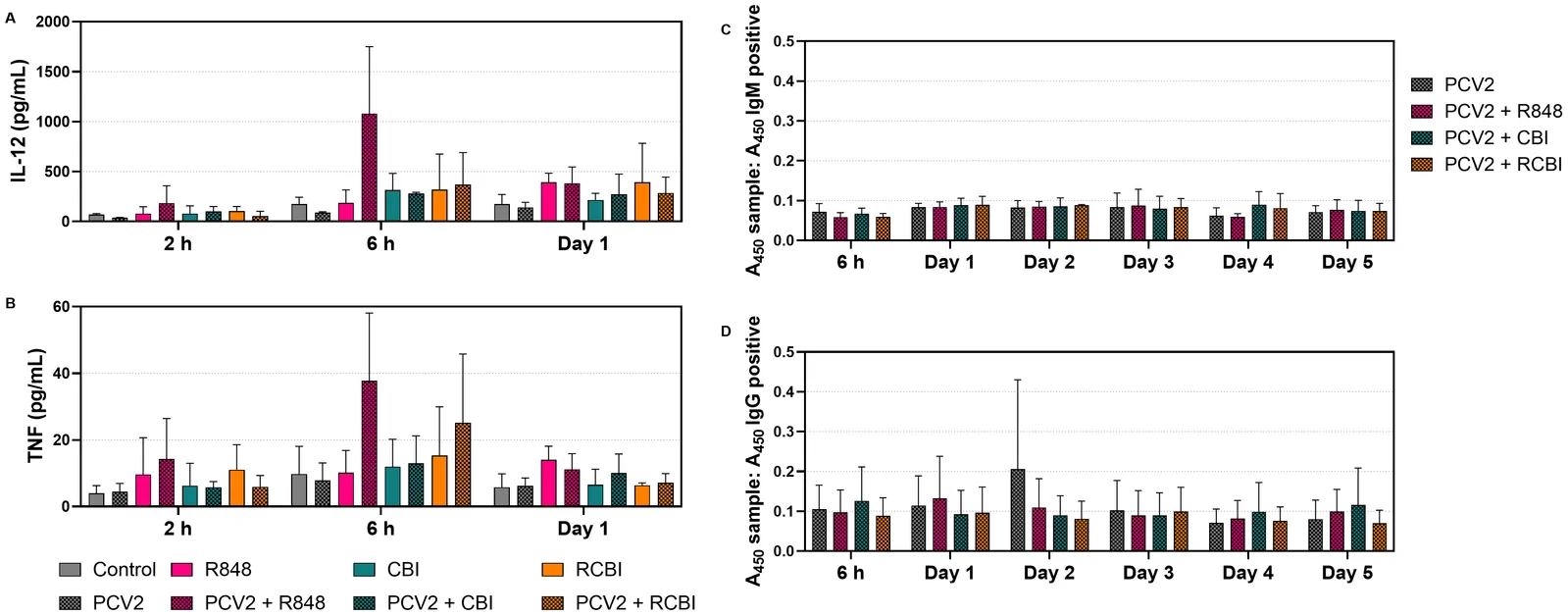
Cytokine production and PCV2-specific antibody responses in PCLNS following stimulation with R848, CBI, PCV2 and their combinations. **(A, B)** Concentrations of **(A)** IL-12/IL-23p40 and **(B)** TNF expressed as pg mL^-1^ in PCLNS supernatants from 2 h to day 1 following treatment with R848, CBI, PCV2 or their combinations. Absorbance was measured at 450 nm (A_450_) and concentrations were interpolated using a 5-PL standard curve. **(C, D)** PCV2-specific **(C)** IgM and **(D)** IgG responses in PCLNS supernatants from 6 h to day 5 following treatment with PCV2 alone or in combination with R848, CBI or RCBI. Antibody responses are presented as A_450_ ratios (sample absorbance/positive control absorbance) to enable inter-experimental standardisation. Data are presented as mean ± SD of *n* = 3 biological replicates measured in technical duplicates. Statistical significance was determined by two-way repeated-measures ANOVA followed by Tukey’s multiple comparisons test.

Based on prior findings from multi-cytokine screening, IL-12 and TNF levels were quantified at 2 h, 6 h and days 1 to 5 (**Figures 8A, B**). As cytokine kinetics plateaued beyond day 1, graphical representation is restricted only up to day 1. A treatment-specific fast induction of both, IL-12 and TNF was observed at 6 h in PCV2 + R848 group. A modest increase in TNF was also detected at 6 h in PCV2 + RCBI condition. No treatment-induced IL-12 and TNF responses were detected in the remaining treatment groups.

PCV2-specific IgG and IgM were measured in all PCV2 treated conditions to determine the ability of the PCLNS to mount a PCV2-specific recall response (**Figures 8C, D**). Overall, PCV2-specific IgG and IgM ratios remained low, indicating the lack of a robust immune response, except on day 2 in the PCV2-alone condition, where a transient IgG peak was detected within the tissue slices.

### Synergistic PCV2 and R848 stimulation elicits a stronger humoral and cytokine response compared to PCV2-VLP and R848 stimulation

**Figure 9:**
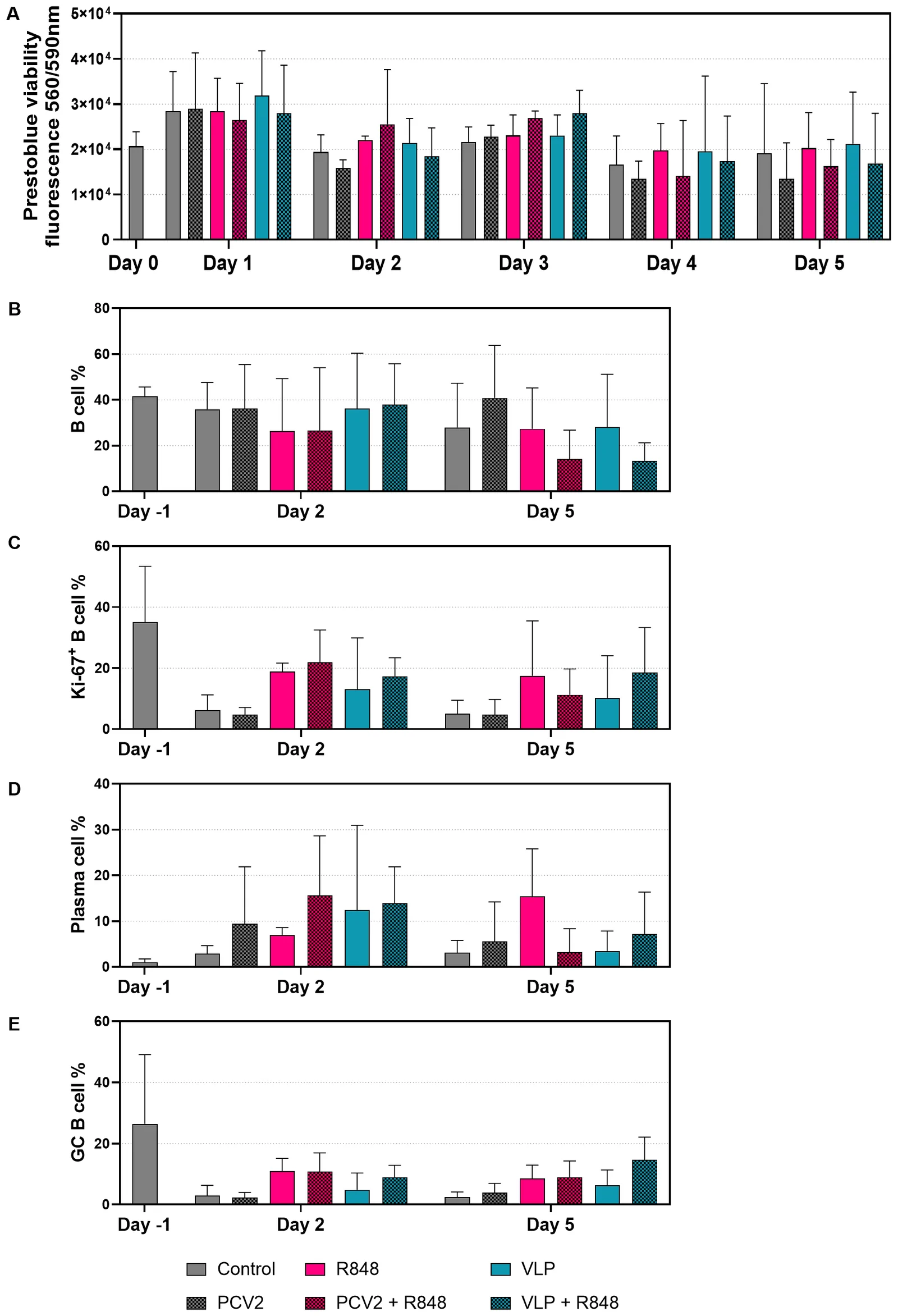
Metabolic viability and B cell phenotypes in PCLNS following stimulation with PCV2, PCV2-VLP, R848 and their combinations. **(A)** Viability of PCLNS was monitored from days 0 to 5 following treatment with PCV2, PCV2-VLP, R848 or their combinations. Metabolic viability was assessed using a resazurin-based assay and measured at excitation/emission wavelengths of 560/590 nm. **(B–E)** Percentages of B cell subsets in porcine PCLNS at day −1 (derived from fresh LN single-cell suspensions), day 3 and day 5 following stimulation with PCV2, VLP, R848 or their combinations. Populations analysed include **(B)** total B cells, **(C)** Ki-67^+^ B cells, **(D)** plasma cells and **(E)** GC B cells. All subsets were identified by sequential gating on FSC-A versus SSC-A, singlets, live cells and the indicated B cell populations. Data are presented as mean ± SD of *n* = 4 biological replicates. For the metabolic activity assay, each biological replicate represents the mean of two duplicate PCLNS, each measured in technical duplicate. Statistical significance was determined by two-way repeated-measures ANOVA followed by Tukey’s multiple comparisons test. The day 0 timepoint was excluded from the metabolic activity analysis and the day −1 timepoint was excluded from the B cell phenotyping analysis.

One of the key features of vaccines is the induction of protection, and organ platforms could play an important role in assessing differences between pathogen and vaccine induced reactions. Thus, to compare immune responses induced by purified virus like particles (VLP) derived from PCV2vaccine-derived PCV2-VLPs and live PCV2, PCLNS were stimulated with either VLPs or PCV2 in combination with R848. Viability of the LN slices increased from day 0 h to day 1, followed by a slight non-significant reduction in viability on day 2 and day 3 (**Figure 9A**), followed by a further non-significant decline on day 4 and day 5 (**Figure 9A**). In addition, no significant differences in viability were observed between any treatment groups (**Figure 9A**).

Baseline B cell proportions were similar across pigs on day -1, whereas pronounced stochasticity within the pigs was observed from day 2 (**Figure 9B**). Overall, B cell proportions were comparable across all treatments on day 2 compared to day -1 control, followed by a marginal decline by day 5 in all conditions except PCV2. Ki-67^+^ B cells declined progressively from day -1 to day 5 in all treatment groups, with lowest levels recorded in PCV2 treated and control slices on day 2 and 5 (**Figure 9C**). R848 (± PCV2 or VLP) and VLP stimulation demonstrated marginally higher Ki-67^+^ B cells at day 2 and 5. Plasma cell proportions increased across all stimulated conditions on day 2 relative to baseline and control, with highest levels observed in PCV2 + R848, VLP + R848 and VLP stimulated groups (**Figure 9D**). Subsequently, reduction in plasma cells was observed by day 5 relative to day 2 in all groups except for R848 stimulated slices. GC B cells were markedly depleted in comparison to day -1 (**Figure 9E**).

**Figure 10:**
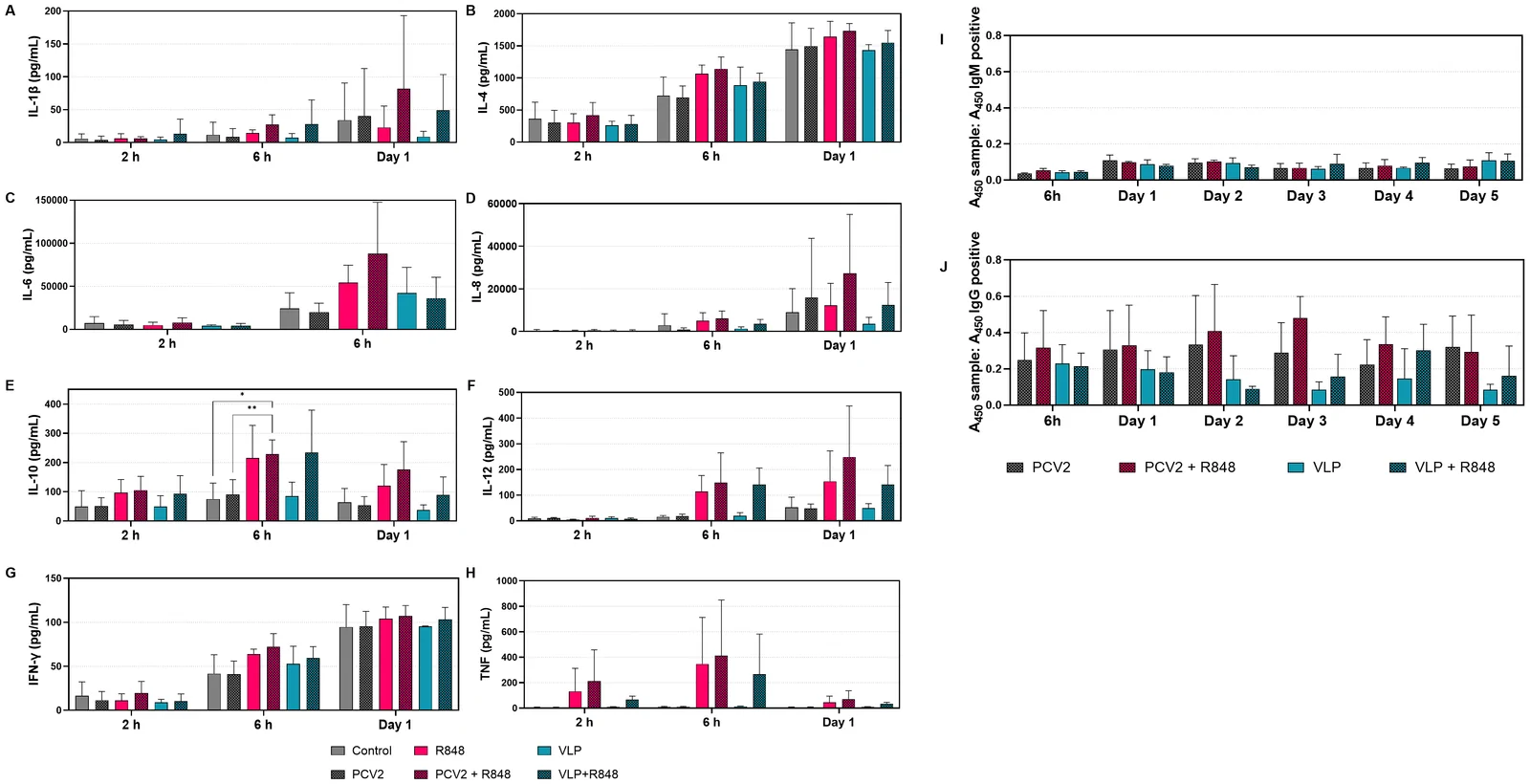
Cytokine production and PCV2-specific antibody responses in PCLNS following stimulation with PCV2, PCV2-VLP, R848 and their combinations. **(A–H)** Time-course analysis of cytokine concentrations in PCLNS supernatants from 2 h to day 1 following treatment with PCV2, VLP, R848 or their combinations. Concentrations of **(A)** IL-1β, **(B)** IL-4, **(C)** IL-6, **(D)** IL-8, **(E)** IL-10, **(F)** IL-12, **(G)** IFN-γ and **(H)** TNF are expressed as pg mL^-1^. Cytokines were quantified using a multiplex bead-based immunoassay, and concentrations were interpolated from 5-PL standard curves generated using Bio-Plex™ Manager software. **(I, J)** PCV2-specific **(I)** IgM and **(J)** IgG responses in PCLNS supernatants from 6 h to day 5 following treatment with PCV2, VLP, R848 or their combinations. Antibody responses are presented as A_450_ ratios (sample absorbance/positive control absorbance) to enable inter-experimental standardisation. Data are presented as mean ± SD of *n* = 4 biological replicates. Statistical significance was determined by two-way repeated-measures ANOVA followed by Tukey’s multiple comparisons test. Statistical significance is indicated as *p ≤ 0.05, **p ≤ 0.01, ***p ≤ 0.001, ****p ≤ 0.0001.

Cytokine screening of culture supernatants showed detectable induction of IL-1β, IL-4, IL-6, IL-8, IL-10, IL-12, IFN-γ and TNF (**Figure 10**). IL-1β increased by day 1 relative to the 2 and 6 h, specifically in PCV2 + R848, followed by PCV2, VLP + R848 and control cohorts (**Figure 10A**). IL-4 increased progressively across all treatment groups from the 2 h to day 1 (**Figure 10B**). IL-6 surged at 6 h, most prominently in PCV2 + R848 treated PCLNS and surpassed the upper quantification limit by day 1, precluding further quantitative analysis (**Figure 10C**). IL-8 remained low at 2 and 6 h, before increasing substantially on day 1 in PCV2 + R848 stimulated group, followed by PCV2-alone treatment (**Figure 10D**). IL-10 peaked at 6 h, with highest concentration recorded across all R848 supplemented conditions including R848, PCV2 + R848 and VLP + R848 (**Figure 10E**). Notably, IL-10 was significantly higher in PCV2 + R848 treatment group compared to PCV2 alone and control at this timepoint. By day 1, IL-10 levels declined in these groups but remained higher than in treatment groups lacking R848. IL-12 was induced by R848, remaining at baseline at 2 h before increasing from 6 h to day 1 (**Figure 10F**). While concentrations increased gradually across all R848-treated groups at 6 h, levels peaked on day 1 in the PCV2 + R848 condition. IL-12 remained at baseline in all non-R848 conditions throughout the culture period. IFN-γ mirrored kinetic pattern to that of IL-4, exhibiting a gradual increase over time irrespective of the treatment (**Figure 10G**). TNF showed rapid induction following R848 stimulation, increasing at 2 h and peaking at 6 h in PCV2 + R848 stimulated group, followed by R848 and VLP + R848 conditions, before declining across all groups by day 1 (**Figure 10H**).

PCV2-specific IgG and IgM levels were measured in the culture supernatants of PCV2 and VLP stimulated PCLNS in presence or absence of R848 (**Figures 10I, J**). No PCV2 specific IgM release was detected under any specific condition over time (**Figure 10I**). There was a tendency for some treatment specific PCV-2IgG responses over time; however, due to the size of the error bars between samples/treatments, none of these differences reached statistical significance. (**Figure 10J**).

## Discussion

The presented research aimed to establish porcine PCLNS as an *ex vivo* model for investigating innate and adaptive immune responses following the use of model antigens including TLR agonist and PCV2. Key requirements for validating PCLNS as a reliable *ex vivo* model included demonstrating the retention of structural organisation, cellular composition and the capacity to mount measurable immune responses during culture. Across experiments, PCLNS remained viable, partially preserved lymphoid architecture and supported plasma cell proliferation, cytokine secretion and PCV2-specific IgG production. However, PCLNS also exhibited inherent limitations including progressive loss of viability and depletion of T cells and GC B cells at later timepoints. These constraints suggest that the current system is best suited for analysing short-term immune dynamics, particularly recall extrafollicular immune response, rather than follicular GC-mediated response, but also showed that further technical issues needs to be addressed.

### Targeting TLR7/8 via R848 seems to induce stronger plasma cell proliferation and cytokine responses in PCLNS

PCLNS maintained structural and metabolic integrity for several days despite slicing-associated trauma, although substantial non-selective cell migration occurred by day 3 across all groups, including controls, indicating that mechanical disruption rather than stimulation drove egress. R848 stimulation altered B cell dynamics, by increasing Ki-67^+^ proliferating B cells and plasma cells (25, 41–43), indicating that B cells can be effectively induced in PCLNS via TLR7/8 activation. Histology confirmed progressive follicular deterioration, with reduced follicle numbers, likely reflecting suboptimal culture conditions rather than physiological GC regression. Further, R848 induced IL-10, IL-12 and TNF. Non-specific IL-1β, IL-6 and IL-8 increases likely reflected tissue stress. R848-induced cytokines were consistent with porcine and human TLR7/8 responses (24, 44) and likely originated from cDC1, B cells, γδ T cells, and macrophages (45–47). Cytokine kinetics suggested immune feedback regulation, with early TNF and IL-12 followed by sustained anti-inflammatory IL-10, which may even promote plasma cell differentiation (48–52). R848 induced robust B cell proliferation, plasma cell expansion and cytokine production, supporting its use as a primary stimulus and its potential to enhance PCV2-specific humoral recall responses.

### B cell survival factors failed to rescue GC responses in PCLNS

Despite strong plasma cell and cytokine responses under R848 stimulation, progressive follicular and GC loss indicated insufficient supportive signalling. GC formation and maintenance typically require multiple co-stimulatory signalling cues provided by cells such as T follicular helper (Tfh) cells, follicular DCs and other stromal populations (53, 54). These signalling pathways encompass CD40L-CD40, ICOSL-ICOS and BAFF-BAFFR/BCMA/TACI interactions, alongside CXCL13-CXCR5 chemotaxis and cytokines such as IL-21, all of which have been primarily described in mice and humans and some in pigs (39, 40, 55–57). Given that CD40L, BAFF, and IL-21 support B cell survival, Tfh development, GC formation and plasma cell differentiation (34, 58–60), these factors (CBI) were supplemented to prolong GC integrity. Despite supplementation, CBI did not prevent decline in viability by day 3 or GC loss by day 5. Histology showed densely populated but GC-negative follicles with areas of parafollicular depletion, suggesting excessive signalling or overstimulation-induced death. PCV2 treated slices early follicular hyperplasia followed by depletion, but classical PCV2 lesions were absent (61–65). While this is consistent with vaccination and qPCR-confirmed absence of active infection, whether PCV2 replicated in the slices remains unclear. Flow cytometry confirmed decline in B cell proportions despite CBI, indicating culture conditions were insufficient to maintain lymphocyte viability. A modest, non-significant rise in plasma cells by day 5 suggests that survival factors and R848 still supported late differentiation. Cytokine analysis showed IL-12 and TNF induction at 6 h exclusively in PCV2 + R848 stimulated slices, indicating synergy between viral antigen and TLR7/8 activation. Notably, treatment with R848, PCV2, or (± PCV2) failed to induce comparable cytokine levels. Despite reports that CBI enhances antigen-specific antibody responses (34, 39, 66–71), only one PCV2 stimulated PCLNS produced detectable PCV2-specific IgG. This variability likely reflects slice-to-slice differences in memory B cell content, LN heterogeneity, and uneven distribution of antigen-experienced cells (72). Collectively, although CD40L, BAFF and IL-21 are central to GC biology, daily supplementation did not improve viability, follicle number, or GC B cell maintenance in PCLNS and further supplementation risks generating an overly artificial system.

### Synergistic PCV2 and R848 stimulation elicits a stronger humoral and cytokine response compared to PCV2-VLP and R848 stimulation

Given the cytokine responses and PCV2-specific IgG were most prominent in PCV2 + R848 stimulated slices, follow-up experiments tested whether PCV2-VLPs could elicit comparable responses with R848. PCLNS viability again declined after day 3, thereby necessitating that experimental analyses be restricted to shorter timeframes. Immunofluorescence identified B and T cell regions, though limited fluorochrome availability restricted additional markers. Phenotyping again showed poor GC B cell maintenance but consistent plasma cell expansion. Concurrently, cytokine profiles under R848 reproduced IL-10, IL-12, and TNF induction.

After the 24h rest period, PCV2-specific IgG appeared as early as 6h and persisted to day 5, declining after day 3. While there is the possibility that this early IgG response is a combination of secretion of already existing and newly formed PCV2-specific IgG antibodies, the absence of PCV2-specific IgM supports a memory-driven recall response, likely extrafollicular given GC loss. A strongest IgG response occurred in PCV2 + R848 slices, indicating that R848 enhances PCV2-specific humoral immunity, consistent with findings in pigs (27, 28), mice (73–75) and non-human primates (76). Notably, PCV2 only slices generated the second-highest PCV2-specific IgG response despite minimal cytokine induction, suggesting cytokine magnitude is not the sole determinant of antibody production. VLP + R848 slices produced lower IgG than PCV2 + R848, contrasting with murine studies where TLR7 stimulation optimised VLP-specific IgG (58). Although VLP input was normalised by particle amount, PCV2 was not titrated, so antigen dose cannot fully explain differences. Stronger responses in PCV2 + R848 slices may reflect greater biologically active antigen exposure, active viral replication and engagement of additional viral sensing pathways beyond capsid recognition (77, 78). These findings present interesting possibilities, implying that targeted TLR7/8 activation of B cells could reactivate memory B cell pools against previously encountered pathogens. This approach could serve as a strategy for broad-spectrum immunity boosting, although this hypothesis remains to be confirmed *in vivo*.

### Limitations and potential ways forward

PCLNS provide a useful *ex vivo* system for capturing early innate and humoral recall responses within six hours of stimulation, but several limitations remain. The model has a short functional window, with tissue viability and follicular structure declining after three days. Efforts to preserve follicular architecture using CD40L, BAFF and IL-21 suggest that the *ex vivo* environment lacks key cues needed to maintain long-term GC activity. New approaches should include further optimisation of the culture medium, embedding slices in biomimetic hydrogels (77, 78) or the incorporation of an artificial capsula, as shown recently in the context of the pleura for human precision cut lung slices (79) may help extend tissue lifespan.

Because slices were collected at fixed timepoints, the dataset reflects independent snapshots rather than true longitudinal dynamics, and natural variation in slice cellularity and LN origin likely increased experimental noise. The analysis focused solely on B-cell phenotyping, preventing confirmation of the exact cytokine-producing populations. Histology showed histiocytes in follicular and parafollicular regions, indicating they likely contributed to cytokine production, but their roles remain inferred. The presence of active PCV2 infection in the slices was not directly verified, though the strong PCV2-specific IgG response after R848 co-stimulation suggests antigen processing occurred. Neutralising antibody responses were not assessed and remain an important future direction.

Overall, this work shows that PCLNS can support short-term recall responses while preserving lymphoid architecture. The platform enables controlled testing of drugs, vaccine candidates, host–pathogen interactions and complex immune cell dynamics *in vitro*. It also allows multiple treatment conditions to be evaluated from a single animal, reducing animal use compared with traditional *in vivo* studies. However, while informative, *ex vivo* systems cannot fully replicate the complexity of whole-organism immunity.

## Data Availability

The datasets presented in this study can be found in online repositories. The names of the repository/repositories and accession number(s) can be found in the article/**Supplementary Material**.
